# DNA Methylation and Non-Coding RNAs during Tissue-Injury Associated Pain

**DOI:** 10.3390/ijms23020752

**Published:** 2022-01-11

**Authors:** Jahanzaib Irfan, Muhammad Rizki Febrianto, Anju Sharma, Thomas Rose, Yasamin Mahmudzade, Simone Di Giovanni, Istvan Nagy, Jose Vicente Torres-Perez

**Affiliations:** 1 Nociception Group, Department of Surgery and Cancer, Division of Anaesthetics, Pain Medicine and Intensive Care, Chelsea and Westminster Hospital Campus, Imperial College London, 369 Fulham Road, London SW10 9FJ, UK; j.irfan18@imperial.ac.uk (J.I.); m.febrianto18@imperial.ac.uk (M.R.F.); anju.sharma19@alumni.imperial.ac.uk (A.S.); thomas.rose16@imperial.ac.uk (T.R.); yasamin.mahmudzade16@imperial.ac.uk (Y.M.); 2 Department of Brain Sciences, Division of Neuroscience, Imperial College London, E505, Burlington Danes, Du Cane Road, London W12 ONN, UK; s.di-giovanni@imperial.ac.uk; 3 Department of Brain Sciences, Dementia Research Institute, Imperial College London, 86 Wood Ln, London W12 0BZ, UK; 4 Departament de Biologia Cellular, Biologia Funcional i Antropologia Física, Facultat de Ciències Biològiques, Universitat de València, C/Dr. Moliner 50, 46100 Burjassot, Spain

**Keywords:** epigenetic, neuropathic pain, nociception, gene transcription, dorsal root ganglion, spinal dorsal horn, miRNA, siRNA, lncRNA, CpG islands

## Abstract

While about half of the population experience persistent pain associated with tissue damages during their lifetime, current symptom-based approaches often fail to reduce such pain to a satisfactory level. To provide better patient care, mechanism-based analgesic approaches must be developed, which necessitates a comprehensive understanding of the nociceptive mechanism leading to tissue injury-associated persistent pain. Epigenetic events leading the altered transcription in the nervous system are pivotal in the maintenance of pain in tissue injury. However, the mechanisms through which those events contribute to the persistence of pain are not fully understood. This review provides a summary and critical evaluation of two epigenetic mechanisms, DNA methylation and non-coding RNA expression, on transcriptional modulation in nociceptive pathways during the development of tissue injury-associated pain. We assess the pre-clinical data and their translational implication and evaluate the potential of controlling DNA methylation and non-coding RNA expression as novel analgesic approaches and/or biomarkers of persistent pain.

## 1. Introduction

### 1.1. Pain as a Disease

The overwhelming majority of lasting pain experiences (pain thereafter for brevity) develop as a response to tissue injury [1]. The tissue injury-associated pain constitutes an adaptive measure, which helps the body to protect the injured tissues from further injuries; hence, it promotes healing [2]. Normally, pain developing after tissue injuries ceases when tissue recovery is completed. Nonetheless, severe injuries are accompanied by intolerable pain, and pain may also become disproportionate in less severe injuries [3]. Furthermore, several tissue damages would not heal and pain may persist even after the healing process is completed [3,4]. In those cases, pain does not serve any biological function and it constitutes a maladaptive measure, a disease *per se* [4].

While severe, disproportionate or persistent pain significantly reduces quality of life, current approaches, which are symptom-based, often fail to control such pain to satisfactory levels [5]. Therefore, we must understand pain-signalling (nociceptive) mechanisms better to be able to develop new mechanism-based analgesic approaches. One of the most novel and promising areas for such analgesic approaches is to control the regulation of transcriptional activities in cells involved in nociceptive processing.

### 1.2. Transcriptional Changes Are Pivotal for the Persistence of Tissue Injury-Associated Pain

The development of tissue injury-associated pain starts with sustained activation, by agents present in injured tissues of specialised nerve cells (nociceptors), a group of primary sensory neurons able to detect pain-inducing stimuli [6]. The resulting barrages of pain signals are then transmitted, as action potentials, to the central nervous system (CNS; [6]). Following processing at various so-called nociceptive areas in the CNS, the signals arrive to multiple encephalic areas where integration of cortical activities leads to the development of the unpleasant experience of pain [2,7]. That unpleasant experience of pain associated with tissue injuries includes hypersensitivities such as allodynia, when physiologically innocuous impacts (e.g., light pressure or the body temperature) induces pain and hyperalgesia, when noxious stimuli induce more intense pain than they do in physiological condition [7].

At the cellular level, the development and persistence of hypersensitivities depend on the most characteristic event in nociceptive processing in tissue injury, a use-dependent increase in the activity and responsiveness of cells (sensitisation) involved in such processing in the nervous system [8,9]. While sensitisation develops in all nociceptive areas of the nervous system, sensitisation of nociceptors and cells in the dorsal horn of the spinal cord, respectively referred to as peripheral and spinal sensitisation, are best characterised [10,11]. One of the main reasons why studies focus on peripheral and spinal sensitisation is that nociceptors and the spinal dorsal horn constitute the preferred targets for novel analgesics; targeting nociceptors promises less undesirable CNS-mediated adverse effects as nociceptors are outside of the blood–brain and blood–nerve barriers, whereas nociceptive processing in the spinal dorsal horn determines which signals can reach supraspinal and cortical areas [9,12].

At the molecular level, sensitisation comprises three mechanistically and temporally different components [6,10]. The first and second components are the immediate and early changes, which involve amplification of responses of ion channels (e.g., transient receptor potential channels (TRP) and acid-sensing channels (ASIC) among others), and posttranslational modifications (PTMs; mainly phosphorylation) of a series of molecules (for further details see [6,11,13]). Those changes occur within seconds and minutes post-injury, respectively. The third component involves transcriptional alterations driving the expression of multiple pro-nociceptive genes (discussed in following sections) which are the most important changes for the persistence of tissue-injury-associated pain [6,10]. Transcriptional changes occur from tens of minutes after the injury.

### 1.3. Epigenetic Mechanisms Regulate Gene Transcription in Adaptive and Maladaptive Responses

According to the “modern” definition, epigenetic mechanisms induce heritable changes in gene function that do not involve changes in DNA sequence [14]. However, studying mechanisms of adaptive changes revealed that the changes induced by epigenetic mechanisms in gene functions are not necessarily heritable. In fact, environmental clues activate epigenetic mechanisms and those mechanisms are pivotal for the generation of a series of long-term responses both in physiological and pathological conditions [13]. Hence, it is not a surprise that epigenetic mechanisms (including histone modifications, DNA methylation and the expression of non-coding RNAs (ncRNA)) regulating transcriptional changes have been found to be activated in neurons and glia cells in nociceptive areas of the nervous system following tissue injuries [1,15]. In fact, epigenetic mechanisms appear to be crucial for regulating genetic programmes responsible for the maintenance of the sensitised state of the cells, hence pain [1,16].

**Figure 1:**
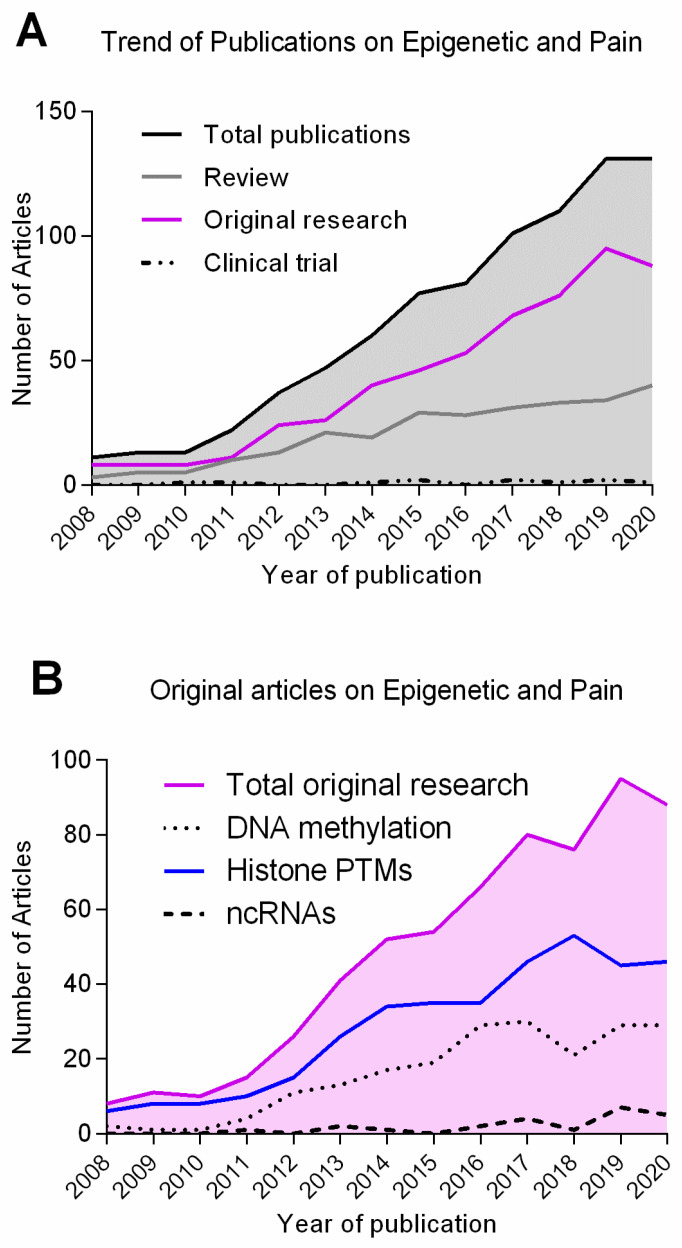
Number of publications on epigenetics and pain from 2008 to 2020. Number of results returned from a search on PubMed (https://pubmed.ncbi.nlm.nih.gov/; access date 15 June 2021) for: (**A**) pain + epigenetic; and (**B**) pain + DNA methylation/histone PTM/ ncRNA. Black: all research papers; grey: review articles; magenta: original research articles; dash-dot patter: clinical trials; dashed line: ncRNAs; dotted line: DNA methylation; blue: histone PTMs. Graphs were generated using software GraphPad Prism Version 7.03.

Although recent years have seen a substantial growth of publications in the field of pain epigenetics (Figure 1), the role epigenetic mechanisms in developing and maintaining prolonged pain remains, to a great extent, elusive. Thus, this review provides a critical evaluation of our current understanding on two types of epigenetic modifications, DNA methylation and non-coding RNA expression, in the development and maintenance of tissue injury-associated pain (outlined in Table 1). Furthermore, we assess DNA methylation and ncRNA expression as potential diagnostic tools, targets for analgesia, and novel approaches to comprehensive elucidation nociceptive mechanisms. A recent review on the role of the third major epigenetic mechanism, histone PTMs, in the development of tissue injury-associated pain can be found in [1].

## 2. DNA Methylation and Pain

### 2.1. DNA Methylation Is Associated with Gene Silencing

**Figure 2:**
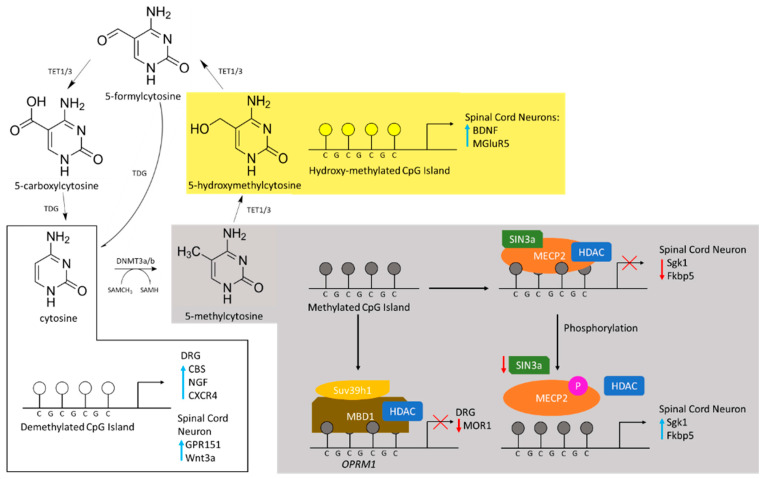
Summary of the cytosine methylation cycle, readers of DNA methylation, and mechanisms of gene expression and inhibition. Demethylated cytosine is methylated by DNMT3a and 3b using the methyl group donated by the cofactor SAM. 5-methylcytosine can be hydroxylated to 5-hydroxymethylcytosine by TET enzymes and then further oxidised to 5-carboxylcytosine. Thymine DNA glycosylase (TDG) may convert 5-carboxylcytosine or 5-formylcytosine back to cytosine. Demethylated CpG islands are associated with the upregulation of various proteins in various pain models (white box). Within the dorsal root ganglia, demethylation is associated with upregulation of CBS, NGF and CXCR4. In spinal cord neurons, demethylation is associated with the transcription of GPR151 and *Wnt3a*. Different preclinical models show pain is associated with methylated CpG islands (grey box). In dorsal root ganglia, methylation may be read by MBD1, which indirectly leads to downregulation of the μ-opioid receptor 1 (MOR1). Under normal conditions within spinal cord neurons, MeCP2 may bind and recruit SIN3a and the HDAC to repress transcription of *Sgk1* and *Fkbp5*. In a CFA model, the binding of MeCP2 is reversed by phosphorylation and leads to the transcription of *Sgk1* and *Fkpb5*, which are associated with mechanical hypersensitivity. TET-dependent demethylation of 5-methylcytosine is associated with the transcription of *BDNF* and *MGluR5* via hydroxyl-methylated promoter regions of spinal cord neurons (yellow box).

The addition of a methyl group (-CH_3_) to the DNA constitutes an inducible mark on the chromatin. The predominant site of methylation on the mammalian DNA is at the cytosine nucleotide [96,97]. Specifically, methylation occurs at the 5-carbon of its pyrimidine ring, resulting in 5-methylcytosine (Figure 2). Cytosines are primarily methylated in the so-called CpG sites or CG sites (CpGs), in which cytosine is followed by a guanine nucleotide in the 5′ → 3′ linear sequence of bases [96,97]. CpG sites tend to form clusters named CpG islands, which are often localised in close proximity of gene promoters. Around 3/4 of protein-encoding genes in humans present this enrichment at their promoters [98]. Methylation at these sites is regarded as a repressive mark, associated with gene silencing, and is considered a key step towards cellular differentiation and fate determination at different tissues, including the nervous system [19,22,23]. In mature neurons, the pattern of DNA methylation can also be changed by environmental cues and cellular insults such as DNA damage [99]. Furthermore, antisense transcription can regulate the state of DNA methylation at the CpG islands, at the gene promoter, and thus affect gene expression [99,100].

The enzymes that add (writers) this epigenetic tag have methyltransferase activity and use S-adenosyl methionine (SAM) as methyl donor [96,97]. Those that write this epigenetic mark afresh to the DNA are called de novo DNA methyltransferases (DNMT) including DNMT3a and DNMT3b. However, DNA methylation is mitotically heritable through other type of methyltransferases, for instance DNMT1, that use the hemi-methylated DNA as their substrate [101].

Proteins with methyl-CpG-binding domain (MBD) exert their reader function by recognising and binding to methylated CpGs. MBD proteins can have other functional domains that allow them to recruit a variety of other epigenetic complexes and remodelling factors, thus leading to chromatin compaction and transcriptional repression. The main group of MBD-containing proteins comprises a family of seven members: methyl CpG binding protein 2 (MeCP2) and MBD1–6, each with different characteristics [102].

DNA methylation was considered to be a stable and irreversible mark. However, a set of different enzymes involving DNMTs themselves, TET (Ten-eleven translocation family) and Gadd45 proteins has been shown to work as removers of this epigenetic mark (erasers, DNA demethylation) in a process not yet completely understood [103]. Recent research has shown that DNA demethylation can be induced by the formation of 8-oxoguanine (8-oxoG): Oxidative stress results in the production of 8-oxoG, which gets repaired by the enzyme 8-oxoG glycosylase-1 (OGG1). Additionally, OGG1 can recruit TET1, the main enzyme demethylating cytosines, to the site of DNA damage and thus trigger DNA demethylation [104]. This active process of demethylation has been observed during developmental stages, adulthood and ageing, including in mature neurons and affecting memory consolidation and neuronal sensitivity [96,105,106,107].

### 2.2. DNA Methylation during Lasting Pain

Many pro-nociceptive genes contain CpG islands and are thus susceptible to DNA methylation. Nonetheless, only newly acquired DNA methylations seem to have a role in the development and maintenance of persistent pain, which occurs in a temporal and tissue-specific pattern [15]. Wang and colleagues found a modest increase of global DNA methylation in the spinal cord two weeks after chronic constriction injury (CCI) of the sciatic nerve, which is used as a model of peripheral neuropathic pain (when the prolonged pain arises from damage/injury of neurons in the somatosensory system including peripheral processes of primary sensory neurons) in rats [17]. The lack of an increase in DNA methylation after sham surgery suggests that changes in DNA methylation are due to CCI. As CCI is associated with the development of pain-related behaviour, in which the spinal cord plays a crucial role, at least a proportion of changes in the CCI-induced DNA methylation must be linked to the development of mechanical allodynia and thermal hyperalgesia in this model.

In contrast to Wang and colleagues’ findings, Garriga and collaborators have reported both up- and down-regulations of methylation in more than 1000 CpG sites in dorsal root ganglia (DRG), which, in addition to other cells, houses the soma of the primary sensory neurons [108]. These changes occur soon after the induction of another type of animal model of peripheral neuropathic pain called spinal nerve ligation (SNL) and persist for at least 3 weeks [108]. However, interpretation of those changes in the methylome should be done with caution, as they might not always correlate with changes at a transcriptional level: methylation changes can happen at any genomic region and not all genes are equally tightly regulated by the CpGs islands [39].

In addition to global changes in DNA methylation, some studies have identified alterations in the expression of specific genes by changes in DNA methylation. Following the plantar injection of rats with complete Freund’s adjuvant (CFA) that induces an inflammatory reaction, of which pain (“inflammatory pain”) constitutes one of its cardinal signs, a significant up-regulation, at both protein and mRNA levels, of cystathionine-β-synthase (CBS), an enzyme involved in pain sensitisation, was detected in DRG [37]. The up-regulation was associated with a lower level of methylation at the CBS’ promoter region [37]. Using the same inflammatory pain model in mice, an increase in DNA methylation at the promoter of the microRNA (see Section 3.3) miR-219 was observed in the spinal cord. This over-methylation results in a reduced expression of miR-219 a day after the injection. Subsequently, an increase in the expression of the calcium/calmodulin-dependent protein kinase II γ (CaMKIIγ), an enzyme important for central sensitisation and chronic pain, was observed [40]. Accordingly, and importantly, treatment with 5′-aza-2′-deoxycytidine, an inhibitor of DNA methylation, resulted in the reduction of mechanical and thermal hypersensitivity [40].

To date, only a few clinical studies have reported DNA methylation changes associated with pain in humans. An increase in DNA methylation at the promoter region of the extracellular matrix protein secreted protein, acidic rich in cysteine (*SPARC*, a protein modulating cell adhesion, proliferation and survival), at the intervertebral discs, was reported in patients with chronic low back pain [42]. Similarly, a recent study has found a positive correlation at the peripheral blood level between the state of DNA methylation at the promoter of the ion channel transient receptor potential ankyrin 1 (*TRPA1*), a gene important for neurogenic inflammation (which describes an inflammatory process due to the activation of immunocompetent cells by agents released from the peripheral terminals of primary sensory neurons upon activation) and preoperative and chronic pain in humans [43]. *TRPA1* methylation state within human blood samples has also been associated with an increased mechanical sensitivity of patients with Crohn’s disease, an inflammatory bowel condition [44].

### 2.3. Writers of DNA Methylation during Pain Consolidation

Preclinical studies have also found associations between DNA methyltransferases and pain processing. Both DNMT3a and DNMT1 were found to be upregulated in the DRG after a peripheral nerve injury. Those methyltransferases seem to specifically target the promoter region of the gene *Kcna2*, which codes for a voltage-gated potassium channel. Silencing *Kcna2* expression leads to decreased voltage-dependent potassium currents and increases excitability in DRG neurons, which in turn leads to spinal cord sensitisation and neuropathic pain symptoms [22,23].

An up-regulation of DNMT3a2 has been found in the spinal cord of adult mice following CFA-injection, which was associated with the induction of *Ptgs2*, a gene encoding for the pain-associated cyclooxygenase 2 enzyme (Cox-2) [27], as well as colony-stimulating factor-1 (CSF1), a secreted cytokine important for microglia activation following injury [24]. Intrathecal injections of recombinant adeno-associated viruses containing short hairpin RNAs targeting Dnmt3a2 mRNA led to reduced thermal and mechanical hypersensitivity during persistent pain states as assessed in the CFA model, while sparing acute inflammatory nociceptive responses [27]. In addition, intrathecal administration of the DNMT inhibitor zebularine either before or after the CFA injection has a dose-dependent effect in reducing thermal hyperalgesia [15].

DNMT3a has also been found to regulate gene expression. DNMT3a up-regulation has been detected in astrocytes of the rat spinal dorsal horn following SNL [24]. DNMT3a also appears to play an important role in the mouse amygdala, and that role might account for differences in pain vulnerability [25].

### 2.4. Readers of DNA Methylation Associated with Lasting Pain

MeCP2 is a reader of DNA methylation with the key function for neuronal maturation. Loss-of-function mutations on its gene are associated with Rett syndrome, a rare neurodevelopmental disorder, mainly affecting females in which enhanced/altered pain perception is a common feature [109]. Binding of MeCP2 to methylated-CpGs is mainly regarded as repressive (silencing) to many genes including brain-derived neurotrophic factor (*BDNF*; important for neuronal development and plasticity), the transcription factor myocyte enhancer factor 2C (*Mef2c*), and ataxin 2 binding protein 1 (*A2bp1*; involved in neurogenesis); although it might also activate transcription of other genes such as somatostatin (*Sst*, encoding a hormone regulating the endocrine system), and opioid receptor kappa 1 (*Oprk1*), which participates in the development of inflammatory hyperalgesia, among others [110,111,112]. However, phosphorylation reduces MeCP2 functions, leading to reduced occupancy at promoter regions [110,113].

Géranton and collaborators were the first to observe MeCP2 changes associated with a rodent model of persistent pain. They found an increase in MeCP2 phosphorylation within neurons in the superficial layers of the spinal cord one hour after a CFA injection [18]. This phosphorylation was linked to the up-regulation of *Sgk1*, a gene encoding a serine/threonine kinase important for long-term memory consolidation, and *Fkbp5*, which regulates glucocorticoid receptors, as well as down-regulation of *Sin3a*, a MeCP2 co-repressor. Further studies from this group have shown that the increase in phosphorylated MeCP2 at the spinal cord following peripheral noxious stimulation is mediated by the serotonergic-descending excitatory system and is implicated in the development of mechanical hypersensitivity [19].

In addition to the global increase in DNA methylation, Wang and colleagues observed an increase in the expression of *Mecp2* at the spinal cord of rats 14 days after CCI [17]. When intrathecally treated with a DNA methyltransferase inhibitor, both mechanical allodynia and thermal hyperalgesia were attenuated [17]. However, another neuropathic pain model, the spared nerve injury (SNI) model that involves transection of the common peroneal and tibial but not the sural nerve [114], resulted in down-regulation of *Mecp2* at both spinal cord and DRG levels [20]. Mice in which *Mecp2* was overexpressed demonstrated an attenuated mechanical and thermal pain sensitivity [21]. These contradictory findings might reflect the different cellular programs associated with different injury types and pathological pain modalities.

MBD1, another reader of DNA methylation, also regarded as a transcriptional repressor, has been implicated in the generation of neuropathic pain at the DRG level. Mo and colleagues reported that MBD1-deficient mice display reduced heat hyperalgesia and mechanical and cold allodynia following SNL, while its overexpression in DRG can rescue these behavioural phenotypes [26]. They have also provided evidence for a role of MDB1 repressing the expression of *Oprm1*, encoding the mu opioid receptor important for pain sensitivity, and *Kcna2* via recruitment of DNMT3 to their promoter regions [23,26]. On the contrary, although MBD4 levels were also up-regulated following CFA injection in rats and were linked to the up-regulation of CBS, the levels of DNMT3a and 3b remained unchanged [37], thus highlighting the context-dependence of those changes.

### 2.5. Erasers of DNA Methylation during Inflammatory and Neuropathic Pain

Local events of DNA demethylation post-induction of different preclinical pain models have also been reported. Although changes in DNA methylation do not necessarily correlate with up- and down-regulation of nociceptive genes, DNA hypo-methylation in relatively few genes might impact the pain phenotype, as observed following nerve injury [39,108]. After intraplantar CFA-injection, a significant demethylation at the promoters of the genes of CBS, nerve growth factor (NGF, facilitates nociceptor response following injury) and the chemokine receptor CXCR4 was detected at DRG neurons [36,57,58], which resulted in their increased expression. Blocking these events led to significant reduction of inflammatory pain hypersensitivity.

Neuropathic pain has also been associated with DNA demethylation in cortical brain areas, which are located away from the site of injury. Six months after inducing a spared nerve injury model in mice, a widespread DNA hypomethylation was observed in the amygdala and prefrontal cortex, which correlated with abnormal sensory thresholds and increased anxiety [115]. Nonetheless, it is not clear whether these changes are pain-related or are involved in other central aspects of peripheral nerve injury.

The expression of GPR151, a G-protein-coupled receptor (GPCR) critical for the maintenance of neuropathic pain, and the chemokine receptor CXCR3, important to recruit immune cells, was up-regulated in spinal neurons after SNL [30,31]. While these up-regulations started three days after SNL and persisted for at least 21 days, transient demethylation at transcriptional starting sites was observed one day earlier and later respectively and lasted until day 9 after the injury. SNL-associated heat hyperalgesia failed to develop, while mechanical allodynia was markedly reduced when *Gpr151* was mutated [31]. Furthermore, intrathecal injection of CXCL10, the ligand of CXCR3, induced pain hypersensitivity in naive mice [30]. A significant decrease in DNA methylation in the promoter region of *Wnt3a* was also observed in the dorsal horn of the rats following the CCI model, thus suggesting that epigenetic regulation of the Wnt-signalling pathway could be critical to the induction and maintenance of chronic pain [32]. As the expression of active β-catenin was also increased, Wnt signalling is postulated to act via its canonical pathway in the dorsal horn of rats following this neuropathic pain model.

TET1, a protein involved in DNA demethylation, has been associated with memory formation in hippocampal neurons [116]. Although preclinical studies did not directly implicate TET1 in nociception, it has a role in sensitised nociceptive states. Spinal TET1-dependent demethylation at the promoter region of the genes metabotropic glutamate receptor subtype 5 (*mGluR5*) and *Bdnf* seemed to mediate neuropathic pain in rats [33,35]. Similarly, mice in which both TET1 and TET3 were knocked down exhibited reduced nociceptive sensitisation when induced by CFA injection, which seemed to result from alterations in spinal expression of the signal transducer and activator of transcription 3 (STAT3) [36]. Overexpression of *Tet1* in DRG can mitigate neuropathic pain through rescuing the expression of *Oprm1* and *Kv1.2* [34].

### 2.6. DNA Methylation as a Therapeutic Target for Chronic Pain and Biomarker of Pain Progression

SAM, the methyl donor used by all DNMTs, has been reported to have analgesic effects. Grégoire and colleagues found that, when administered during the chronic period, it could reduce mechanical hypersensitivity in a murine SNI model [117]. However, SAM’s analgesic effect might be achieved via other mechanisms. For instance, through regulating the activity of the enzyme catechol o-methyltransferase (COMT), which also uses SAM as methyl donor and has been implicated in pain perception [118].

5-aza-2′-deoxycytidine, a DNMT inhibitor currently licensed as a chemotherapeutic, may also be useful as an analgesic based on its efficacy to reduce the levels of MeCP2, as reported by Wang and co-authors [17]. However, the analgesic effect is only achieved when administered intrathecally, thus limiting its potential therapeutic use. Furthermore, other proteins that are potential targets to (de)methylation via the same enzymes might also impact their therapeutic potential. On the other hand, several studies have also reported that reducing DNA methylation can cause pain hypersensitivity as the genes and/or location of the CpG sites (promoters vs. intronic regions) affected will impact differently on the change in gene expression [108]. Thus, DNMT inhibitors could be problematic therapeutic agents.

Analysing the differentially methylated regions from circulating blood has proven useful in the clinic to identify pain biomarkers [46], to differentiate methylome signatures in different types of chronic pain [45], and to identify differential pain sensitivity [47]. However, excluding some exceptions [47], most methylome changes assessed in clinics are performed from circulating samples (e.g., blood) while the nociceptive pathway is not accounted for. Thus, those changes, which can also differ between pain modalities, might not reflect those occurring in tissues directly relevant to pain sensitivity. To complicate things further, ageing is associated with an increase in, among others, reactive oxygen species, which can directly increase DNA demethylation and impact on neuronal processes [106,119]. Hence, it is difficult to standardise the methylome for its use as a biomarker for diagnosis and prognosis. Currently, there is one clinical study aiming to assess the degree of DNA methylation in blood samples of patients undergoing major surgery [120].

### 2.7. Future Directions to Study DNA Methylation during Pain Progression

There are three main techniques which can be used to assess DNA methylation (Table 2): sodium bisulfite conversion and sequencing, differential enzymatic cleavage of DNA, and affinity capture of methylated DNA. Although their use in pain research is sparse, some research has shown its potential in pain and related fields.

Sodium bisulfite conversion and sequencing works by inducing de-amination exclusively to non-methylated cytosines to produce uracil, which, when amplified by PCR, will be converted to thymine. By comparing the profile of this DNA sequence to their non-methylated original, the methylome is inferred [121,122]. Current variations include sequence-based analysis, interaction-based analysis and analysis based on melting temperature of the bisulphite-treated DNA. However, a high DNA yield, as well as additional controls, are needed, since the technique requires extreme temperatures and pH levels that can lead to significant DNA fragmentation. Gölzenleuchter and colleagues used this technique to identify genome-wide methylation changes at DRG of rats one day after the induction of a SNL which in some cases corresponded with changes at a transcriptional level [39]. Clinically, it has been used to identify blood biomarkers of inflammation and bone maturation in individuals suffering from chronic lower back pain [46] and the similarities (neuro-musculoeskeletal genes) and differences (opioid and GABAergic systems) between chronic nociceptive/neuropathic pain [45].

The limitations of bisulfite conversion have driven the development of other approaches to map the methylome, including enzymatically driven differential cleavage of the DNA, such as methylation-sensitive restriction enzymes (MREs) and digital restriction enzyme analysis of methylation (DREAM). MREs can selectively cut the non-methylated CpGs while sparing the methylated CpGs. Fragments are then size-selected and sequenced to reveal the location of non-methylated sites [129]. However, since only around 20% of the CpG islands are dynamically regulated, MRE-sequencing lacks efficiency genome-wide and is more suited for specific loci [130]. A luminometric variation of this assay (LUMA: Luminometric Methylation Assay) has been used to show a decrease in global DNA methylation in both the PFC and amygdala following six months of peripheral nerve injury in mice [115]. LUMA combined with sodium bisulfite has shown the implication of individual genes, including *syt2* (synaptotagmin 2), which regulates Ca^2+^-dependent neurotransmitter release [41]. DREAM, on the contrary, offers a high resolution at the genome level. It induces methylation-specific signatures at the end of DNA fragments and their posterior analysis by next-generation sequencing [127]. In the context of chronic pain, DREAM has been successfully used to demonstrate the contribution of DNA methylation to the reprograming of DRGs [108].

Another way to circumvent issues with previous techniques is to use methods of affinity capture of methylated DNA. Methylated DNA immunoprecipitation followed by sequencing (MeDIP-Seq) allows for high resolution mapping at the genome-wide scale and requires smaller yields (down to 1 ng per sample) [131]. In MeDIP-Seq, the DNA is sonicated, denatured into single strands, and selectively precipitated with specific antibodies. Following DNA amplification, samples can be analysed by next-generation sequencing methods. Similarly, the properties of some proteins with MBD can be used to highlight differentially methylated regions of double-stranded DNA: DNA is sonicated into random fragments, incubated with MBD proteins previously coupled to separation beads, and fragments are then stepwise eluded in a series of differential CpG densities and processed by next-generation sequencing. However, MBD-based techniques have their own limitations [132,133] that make them biased towards hypermethylated regions, whilst MeDIP-Seq does not have this bias and may be used on a wider scale. Massart and collaborators have used this approach to assess differential methylation profiles at both PFC and immune cells (T cells) of rats following SNI [38]. Additionally, using MeDIP-Seq in blood samples from monozygotic twins and the general population, Bell and colleagues identified differential methylation at the promoter region of the ion channel gene *TRPA1*, among others, in association with differential pain sensitivity [47].

Currently, there are other techniques being developed, including polypyrrole guided (PPyox) Multi-Walled Carbon Nanotubes (MWCNTs) immobilised onto a Choline monolayer-modified Glassy Carbon Electrode (Ch/GCE), which uses the direct electrochemical oxidation of DNA [134], or others using High-Performance Liquid Chromatography (HPLC, [135]), to infer the state of DNA methylation. All these techniques, coupled with novel and more powerful computational methods [136], could offer significant advances to study the landscape of DNA methylation in pain research.

## 3. Non-Coding RNAs and Pain

### 3.1. Types of Non-Coding RNAs, Their Function and Classification

A ncRNA is a functional RNA molecule that is transcribed from a portion of DNA, an RNA gene, but is not translated into proteins [137]. Some types of ncRNAs play a direct role in protein translation, such as transfer RNAs (tRNAs) and ribosomal RNAs (rRNAs). There are other ncRNAs that, instead, have a regulatory role by influencing either the state of chromatin, directing DNA methylations and histone PTMs, or the stability of other RNA molecules (e.g., mRNAs). This second type, which has an epigenetic role, can be divided by their size into short ncRNAs (less than 30 nucleotides) and long ncRNAs (lncRNAs; more than 200 nucleotides). Short ncRNAs are further subdivided into three major classes according to their mechanism of action and origin: microRNAs (miRNAs), which base-pair with mRNA complementary sequences to silence them and derivate from the catalysis of a single-stranded RNA by the enzyme Dicer; short interfering RNAs (siRNAs), with a similar function as miRNAs and a similar catalytic origin but deriving from longer regions of double-stranded RNA; and piwi-interacting RNAs (piRNAs), which are involved in gene silencing via RNA-protein interactions and have a different origin [138]. Within lncRNAs, there is a special category called circular RNAs (circRNAs). In contrast to linear RNAs, circRNAs form a covalent link between the 3′ and 5′ ends which generates a continuous loop or circle [139], which confers them with increased stability. CircRNAs are mainly localised at the cytoplasm and have been involved in different molecular mechanisms. The expression of all those epigenetic ncRNAs can be influenced by environmental cues, such as inflammatory mediators, and thus contribute to the progression of pathological conditions including cancer, neurodegenerative disorders and pain signalling [61,76,140,141]. However, interpretation of those changes in expression requires caution as different ncRNAs may have diverse, or multiple, functions, which might still be unknown [142] or, on the contrary, do not serve any biological function [137].

Mitochondria, the organelles in charge of generating energy for mammalian cells, have their own DNA (mitochondrial DNA, mtDNA). The expression of mtDNA can also be regulated by epigenetic mechanisms, including DNA methylation and ncRNA, in what is now termed mitoepigenetics [143]. Reciprocally, the genetic material from the mitochondria can communicate and influence nuclei expression [143]. Recent studies have linked mtDNA’s genetic polymorphisms with different chronic/neuropathic pain conditions [144,145]. Although those polymorphisms are directly linked to defects in oxidative responses, they might influence tissue injury-associated pain. Overall, mitoepigenetics might be a new direction for studies on the role of epigenetics in the development of tissue injury-associated pain.

### 3.2. lncRNAs and circRNAs during Neuropathic Pain Processing

An increasing amount of research has shown that around 1/3 of all described lncRNAs are specifically and dynamically expressed in the nervous system and can mediate complex behaviours. Abnormal expression of lncRNAs has been described in the injured nerves, DRG, spinal cord, hippocampus, and prefrontal cortex of preclinical models of neuropathic pain. Although still not completely understood, dysregulated lncRNAs seem to contribute to the pathogenesis of neuropathic pain (reviewed elsewhere: [91,92,93]). However, all these data come from cellular or animal models, which challenges the translation of these findings to the clinic.

**Figure 3:**
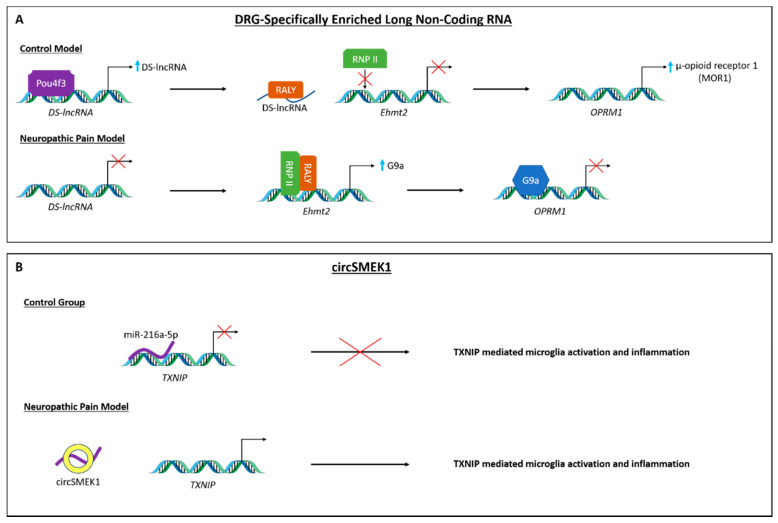
Mechanisms of long non-coding RNA and circular RNA in pain-associated transcription pathways. (**A**) Transcription of a DRG-specifically enriched lncRNA (DS-lncRNA) is promoted by activity of *Pou4f3*. DS-lncRNA inhibits the expression of *Ehmt2* by negative regulation of the RALY/RNPII complex and enables the transcription of μ-opioid receptor 1. In peripheral nerve injury, *Pou4f3* expression is silenced, resulting in the loss of DS-lncRNA transcription which leads to expression of the *Ehmt2* gene. Subsequently produced G9a silences the expression of the μ-opioid receptor, causing nociceptive hypersensitivity. (**B**) MicroRNA-216a-5p inhibits the expression of pro-inflammatory protein TXNIP in microglia. Circular RNA SMEK1 increases the expression of TXNIP by competitively binding miR-216a-5p, thereby promoting neuropathic pain.

Recently, Pan and colleagues have identified a DRG-specifically enriched lncRNA (DS-lncRNA), a lncRNA specifically and highly (>70%) expressed in DRG neuronal nuclei that gets downregulated following different models of peripheral nerve injury (CCI and SNL, [49]). Downregulation of DS-lncRNA, which seems to be mediated by silencing of the *Pou4f3* gene (transcriptional activator POU domain, class 4, transcription factor 3), leads to alterations in the opiodergic signalling system and negatively regulates the expression of RALY-triggered Ehmt2/G9a (Figure 3A) [49]. Ma and colleagues have shown that the lncRNA MALAT1 (metastasis associated lung adenocarcinoma transcript 1) increases at the spinal cord of rats subjected to CCI and seems to contribute to the development of neuropathic pain by downregulating the expression of the miRNA miR-129-5p which, in turn, leads to the up-regulation of HMGB1 (High mobility group box 1; associated with chromatin re-structuring) [50]. Using the same model, Li et al. have shown an upregulation of DLEU1 (deleted in lymphocytic leukemia 1), which seems to silence miR-133a-3p and thus upregulates the expression of SRPK1, a serine/argenine kinase previously implicated in nociception [48]. Xian’s group has shown an increase in the lncRNA NEAT1 (nuclear paraspeckle assembly transcript 1) in rats following spinal cord injury-induced neuropathic pain, which is involved in homeostasis and inflammation via a pathway regulating the activity of microglia [51]. In fact, inhibiting this pathway increases both mechanical paw withdrawal latency and paw withdrawal threshold, the metrics used to ascertain neuropathic pain in this model.

circRNAs have also been involved in neuropathic pain (reviewed elsewhere: [94]). Recent preclinical studies using the CCI model in rats have identified imbalances in different circRNAs. There is an increase in circSMEK1 (Figure 3B), which seems to silence the expression of the miRNA miR-216a-5p and thus contributes to the response of microglia cells [54]; and circ_0005075, which seems to regulate neuropathic pain progression by indirectly (via silencing miR-151a-3p) upregulating notch receptor 2 (*notch2*) [53], known to regulate cell cycles. On the contrary, cZRANB1 is downregulated, which seems to be silencing miR-s4-3p which, in turn, modulates the Wnt/β-Catenin pathway [57].

Furthermore, circRNAs have recently been shown to be involved in cancer pain. In a rat model of bone cancer pain, different circRNAs have been identified as being differentially expressed at the spinal cord. Among those, up-regulation of circSlc7a11, which seems to modulate proliferation and apoptosis through multiple mechanisms, including chemokine signalling pathway, in the murine cell LLC-WRC 256 [56].

### 3.3. miRNAs in the Progression of Both Inflammatory and Neuropathic Pain

miRNAs are, by far, the most widely studied type of ncRNAs in pain research [95]. Differential expression of various miRNAs have been directly reported at DRG after the induction of inflammatory and neuropathic pain, including down-regulation of miR-10a, -29a, -98, -99a, -124a, -134, and -183 in the trigeminal ganglia within 4 h of a CFA injection into the rat masseter muscle [61] or up-regulation of miR-21 following peripheral nerve injury [76]. Additionally, miRNAs have also been indirectly linked to pain progression via molecules that mediate their production and functioning. Zhao and co-authors demonstrated that specific deletion of Dicer in nociceptive primary sensory neurons led to reduced inflammatory pain but not noxious stimulation-induced physiological pain. Nociceptor-specific transcripts were down-regulated, while the expression of other genes was maintained or increased following the CFA injection. This suggests that there is a subset of nociceptor-specific miRNAs, which are involved in the balance of pro- and anti-nociceptive genes being expressed during inflammatory pain [146].

**Figure 4:**
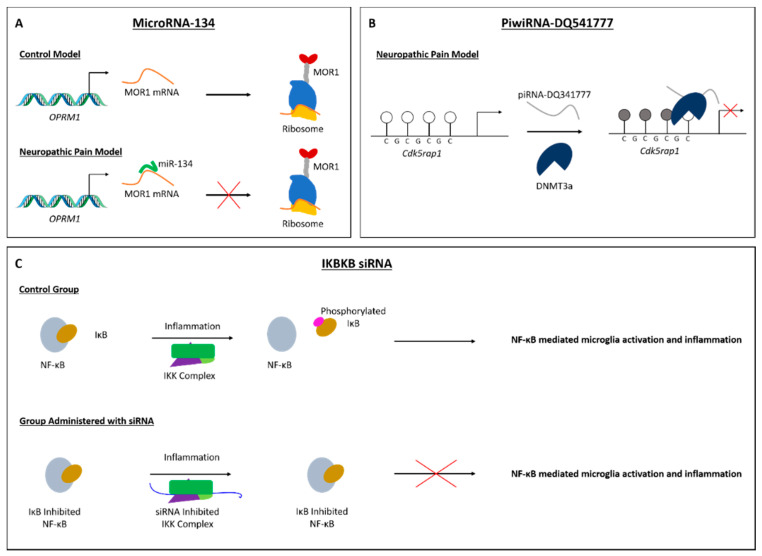
Mechanisms of non-coding RNA in pain-associated transcription pathways. (**A**) Translation of μ-opioid receptor 1 mRNA can be inhibited by the binding of miRNA-134 in a model of inflammatory pain. (**B**) PiwiRNA-DQ541777 is a repressor of the expression of the *Cdk5rap1* gene, contributing to the modulation of neuropathic pain. The piRNA acts via the recruitment of DNMT3a, which silences the target gene by methylating its promoter region. (**C**) Inhibitor of kappa B (IκB) disables the activation of pro-inflammatory NF-κB. In a pain model, phosphorylation of IκB by IKBKB (labelled IKK complex) releases NF-κB, enabling its activity. Inhibition of IKBKB by a siRNA prevents the phosphorylation of IκB, and so NF-κB activity is precluded.

Further studies have established causal links between the imbalances in miRNAs and specific changes in gene expression in preclinical models of both inflammatory and neuropathic pain. Following CFA-induced inflammatory pain, increase in miR-134 expression has been reported in DRG neurons, where it acted as a repressor for *Oprm1* expression, encoding the mu opioid receptor (Figure 4A) [69]. Furthermore, the CFA model of inflammatory pain is also associated with increased miRNA-107 expression in the spinal cord, where it controls the expression of the glutamate transporter 1 (GLT-1) [64]. Decreases in miRNAs have also been reported in the CFA model of inflammatory pain, including miR-485-5p at the DRG, where it seems to up-regulate the expression of the ASIC channel 1 (*asic1*) [86].

Changes in the expression of certain miRNAs have also been reported during early-to-late stages of neuropathic pain, including, among others, the downregulation of miR-140, which could be influencing sensitisation changes via sphingosine-1-phosphate receptor 1 (S1PR1) [72]; miR-216a-5p, influencing neuroinflammation [77]; miR-30a-3p, which indirectly leads to an up-regulation of BDNF [80]; miR-96 and miR-7a, which led to the increased expression of the voltage-gated sodium channel alpha subunit Na_v_1.3 [89] and the β2 subunit of the voltage-gated sodium channel [88]; and miR-590-3p, which has been implicated in a model of diabetic peripheral neuropathic pain and seems to regulated the infiltration of immune cells into the neural tissues [87]. Furthermore, abnormal expression of multiple miRNAs, which target genes involved in nerve regeneration, was observed in DRG and in the proximal stumps of the nerves following rat SNI [147].

A similar association between miRNAs and cell-type-specific transcriptional changes has also been reported in both neurons and glia cells of both neuropathic and inflammatory pain models. In DRG neurons of rats subjected to CCI, there is a concomitant increase of miR-137 with a decrease in the expression of voltage-gated potassium channel Kv1.2, which regulates neuronal excitability [71]. In a murine diabetic peripheral neuropathic model, the expression of miR-33, miR-380 and miR-124-1 has been recently identified in nociceptive neurons [66]. The induction of inflammatory pain by either CFA or formalin injection significantly reduced the expression of miR-219 and miRNA-124a in murine spinal neurons, which negatively regulates the expression of spinal CaMKIIγ [40] and the MeCP2 proinflammatory marker [67]. A decrease in the expression of miR-103 was also observed in spinal neurons of SNL rats, which seemed to simultaneously regulate the translational levels of the three subunits forming Cav1.2-comprising L-type calcium channel (Cav1.2-LTC), involved in pain sensitisation [62]. Similarly, there was a reduction in miR-214-3p in the spinal astrocytes of rats following SNL, which led to their overactivity via the up-regulation of CSF1 [24]. Furthermore, the changing profiles of miRNAs in microglial cells seem to suggest that those cells play different roles depending on the tissue and/or the stage of the pathology (reviewed elsewhere for spinal cord: [148]).

A dysregulation of miRNAs has also been observed in the prefrontal cortex of mice following a carrageenan injection. Poh, Yeo and Ong reported a bilateral increase of miR-155 and miR-223 in the prefrontal cortex of mice treated facially with carrageenan, which correlated with down-regulation of CEBP-beta, a transcription factor important for the regulation of genes involved in immune and inflammatory responses, but up-regulation of granulocyte colony-stimulating factor (GCSF) [74]. The authors suggested that this epigenetic mechanism could lead to an increased inflammation-derived activation of the prefrontal cortex.

The expression of different miRNAs has also been detected in pre-clinical models of cancer-induced pain. In a model of bone metastatic pain, in silico analyses identified 57 miRNAs dysregulated in sensory neurons and predicted several pronociceptive genes as their targets, including *Clcn3*, a gene encoding a chloride channel [149]. In addition, miR-34c-5p has recently been identified in DRG neurons as pronociceptive by regulating the transcription of *Cav2.3*, *P2rx6*, *Oprd1*, and *Oprm1* [84]. In the spinal cord, following the induction of various models of cancer-induced pain, imbalances on the levels of miRNAs have been found: down-regulation of miR-124, which negatively regulated synaptopodin (*Synpo*), a key component in synaptic transmission [65]; up-regulation of miRNA-330, which seems to modulate the expression of GABA_B_R2 [83]; and decreases in miR-135-5p, which regulated JAKs/Stat3 signalling, in spinal astrocytes [70]. Furthermore, changes in miRNAs have also been associated with certain drug-induced pain disorders, such as that induced by the use of oxaliplatin to treat advanced colorectal cancer [150].

### 3.4. piRNA and siRNA in Neuropathic Pain

To date, only one piRNA has been implicated in pain processing. Zhang and colleagues demonstrated that spinal piRNA-DQ541777 (piR-DQ541777) was significantly increased in mice subjected to CCI-induced neuropathic pain which indirectly, via recruiting DNMT3a, repressed the expression of CDK5 regulatory subunit-associated protein 1 (*Cdk5rap1*, Figure 4B) [90].

On the contrary, siRNAs have not been directly implicated in nociceptive processing. However, several studies have looked at their use as potential novel analgesics (reviewed elsewhere: [95]). Recently, Peng’s group has shown that intrathecal administration of an siRNA aiming to silence the lncRNA Uc.48 has therapeutic value in a preclinical model of HIV-associated neuroinflammatory pain [151]. This treatment was able to reverse both mechanical and thermal hyperalgesia in rats previously administrated with the human immunodeficiency virus envelope glycoprotein 120 (gp120). The analgesic effects seems to be a consequence of indirectly inhibiting the upregulation of P2Y12 receptors at their DRG [151].

Similarly, Lee and colleagues have shown that intrathecal delivery of an siRNA against the gene *IKBKB* (inhibitor of NF-κB kinase subunit beta) was able to reduced mechanical allodynia and secretion of pro-inflammatory mediators in rats following the SNL model (Figure 4C) [152]. This research represented a significant advance by incorporating the delivery of poly (lactic-co-glycolic acid) (PLGA) nanoparticles as carriers for the siRNA, which enhanced microglia targeting and thus achieved greater cell-type selectivity.

### 3.5. Could ncRNAs Be Potential Targets/Biomarkers for Pain Management?

NcRNAs can regulate gene transcription by (in) directly affecting the expression of multiple pain-related genes, thus suggesting their use as potential therapeutic targets. For example, Li and colleagues demonstrated that upregulating the expression of a single miRNA, miR-375, could modulate the morphine-dependent analgesic effect and its tolerance [85]; or the use of siRNA as novel analgesics (see Section 3.4). However, one single ncRNA could simultaneously regulate the expression of non-related genes or, additionally, modulate the activity of other epigenetic motifs or their effector enzymes, including DNMTs and histone deacetylases [1,44,125,126]. Therefore, a potential treatment with ncRNA, or to silence a certain ncRNA, should be directed to a specific population within a pain-relevant tissue if aiming to minimise toxicity or side-effects [153]. Strategies such as the use of PLGA particles (mentioned above) that have shown selectivity and efficacy for a neuropathic pain model [152] are worth investigating further. Nonetheless, their therapeutic potential remains narrow as efficacy of tackling ncRNAs has only been proven in preclinical studies via intrathecal administration [44,93,119,126,136,137,140] which, additionally, seems to lead to contradictory findings [154].

Most ncRNAs are capable of travelling long distances throughout the body and thus could serve as biomarkers for the state of a certain pathology, including different pain conditions [95]. A clinical study using plasma from patients affected by diverse types of inflammatory or neurogenic chronic musculoskeletal pain has reported different profiles of circulating miRNA depending on the neuropathic or inflammatory origin of their painful condition [155]. In patients with trigeminal neuralgia, this strategy has served to identify four different miRNAs associated with the occurrence and development of this type of neuropathic pain [68]. A similar strategy has served to identify an association between the upregulation of miR103a/107 and adaptive coping in patients suffering from fibromyalgia [63], and to establish a link between vitamin D deficiency, altered expression of circulating miRNAs and lower back pain in older adults [156]. Currently, there are two clinical studies investigating the profiles of miRNAs as diagnostic biomarkers of pain intensity in adolescents with chronic fatigue syndrome [157] and sleep dysregulation and pain prior to surgery [158].

Nonetheless, the different methods (see below) and standardising procedures (e.g., using serum vs. white blood cells), validity of preclinical methods or variations on the inclusion criteria used for clinical studies, might lead to contradictory findings and thus hamper their translational potential to clinical diagnostics. In that regard, Tramullas’s group has recently identified miR-30c-5p in neuropathic pain by looking at both a preclinical model and patients with ischemia-induced pain [82]. They found an up-regulation of this miRNA, which seems to inhibit the expression of TGF-β, a known mediator of nociception, at the spinal cord, DRG, cerebrospinal fluid (CSF) and plasma of rodents subjected to SNI, and in CSF and plasma from patients. Similarly, two recent publications have studied the mechanism by which down-regulation of miR-183 and up-regulation of miR-30b-5p influence osteoarthritis pain by systematically looking at both a preclinical model and a clinical setting [75,81]. In both cases, reconstituting their expression to pre-injury levels ameliorated the painful condition in the murine model. Comparative studies like these ones might help to validate preclinical finding and to better characterise pain aetiology and thus lead to a more tailored clinical management.

### 3.6. Novel Approaches on ncRNAs for Pain Processing

Elucidation of the function of ncRNAs in pain progression remains challenging. Appropriate techniques should take into considerations their tissue specificity, small size, low level of expression and, importantly, their poor sequence conservation [159].

The microarray-based methods have been routinely used as an initial exploratory tool for ncRNA expression, until high throughput sequencing became widely accessible. Microarrays can detect multiple small size oligonucleotide by using large numbers of specific oligonucleotides known as probes, placed on a solid surface followed by hybridisation of target sequence, and then visualised by fluorescence detection [160]. However, microarrays use already known sequences, thus making them unsuitable for the discovery of novel ncRNAs. Nonetheless, microarray-based methods can be used as cost-effective procedures to validate the presence of ncRNA from a known or predicted transcript and have proven useful in pain research [66,70].

High throughput RNA sequencing (RNA-Seq) methods are preferable to discover novel ncRNAs. They offer a higher degree of specificity and sensitivity and have recently become more accessible and cost-effective. With this technique, Xiong and colleagues were able to identify a total of 374 circRNA differentially expressed in the DRG of rats following the induction of neuropathic pain by CCI (preprint here: [55]). This approach has also been used to identify differentially expressed miRNA, lncRNAs and circRNAs in the spinal cord of a model of diabetic neuropathic pain [59]; lncRNAs and mRNA in the spinal cord of rats subjected to paclitaxel-induced peripheral neuropathy [52]; and miRNA and mRNA in the DRG of rats between a CCI group and a CCI-exercise group [73]. However, RNA-Seq techniques may suffer from PCR-related bias, including library preparation and sequencing errors [161]. Additionally, the nature of several ncRNAs, especially those with less than 200 nucleotides, makes it difficult to detect them by standard RNA-Seq techniques. To overcome this limitation, a small RNA-Seq has been developed [162]. This varies from standard RNA-Seq in employing electrophoresis-based fractionation to specifically separate small RNA, followed by universal adapter ligation on both ends of the RNA fragment and posterior amplification. Using small RNA-Seq, Dai’s group has identified 33 upregulated and 39 downregulated miRNAs used at L3-L6 DRG of rats that were subjected to the SNI model [60]. Moreover, Zhou and colleagues discovered 134 lncRNA, 12 miRNA and 188 circRNA in the rats’ spinal cord following spinal nerve injury [58]. Despite its specificity, small-RNA-Seq may still suffer from adapter ligation bias, which leads to reverse transcription or amplification bias [163].

Although not used to assess the ncRNAs in the pain field yet, there are novel techniques worth considering for future applications: single cell RNA sequencing, with the potential to assess the specificity of ncRNA expression within individual cells at a higher sensitivity/resolution [164]; or nascent RNA sequencing, which can be used to measure changes in RNA state in response to different stimuli [165], including pain. Assessing the changes happening at the single-cell level in all the relevant cellular types (e.g., neurons and glia cells) could help to elucidate how they participate and communicate with each other to orchestrate the entire response, including pain-to-tissue injury. Additionally, the field would benefit from the development of more powerful in silico tools (e.g., http://www.targetscan.org/vert_80/; date accessed: 25 October 2021) which are less dependent on experimental bias and, in turn, can later be tested in the lab. Developing novel ncRNA datasets, similar to those already available (https://lncipedia.org/; http://bioinfo.life.hust.edu.cn/LNCediting/; http://circatlas.biols.ac.cn/; https://www.mirbase.org/, among others; date accessed: 25 October 2021), but specific for pain progression, will help generate a greater insight into their implications for this process.

## 4. Final Considerations and Future Directions

Studying and interpreting how the methylation state of the DNA influences gene transcription during pain progression and consolidation, as well as how ncRNAs regulate this process, should be subjected to the same criticism as that of other types of epigenetic modifications [1], including: misinterpreting correlations by causal relationships, cell/tissue-specificity issues, context-dependence cellular ageing, possible influences from confounding factors and other physiological process (e.g., wound healing) or the combined effect of multiple types of epigenetic tags (e.g., multiple types of ncRNAs [58]). Importantly, other molecular processes might also be at play which could be influencing the epigenetic landscape of pain progression; for instance, a potential mitoepigenetic control [143] or the fact that ncRNAs might also be subjected to epitranscriptomic regulation [166]. Additionally, as stated in previous sections, current experimental techniques used to assess both methylated DNA and ncRNAs can lead to biased results, which should also be taken into account when attempting to interpret the data.

During the consideration of translating those findings to the clinic, one must be aware that some of the techniques that are considered standard practice in the lab might not be feasible to use for clinically relevant samples. For example, while single cell/nuclei RNA-seq can be of use in animal models, it might not seem suited for the same procedure when performed from post-mortem human samples [167]. Therefore, techniques which have provided reliable and high quality data with both patient-derived and preclinical samples in the context of pain [75,82] should become the standard.

The number of studies aiming to target DNA methylation or ncRNAs during different pain paradigms is rapidly increasing and seems to demonstrate their potential as novel analgesics. However, currently, the most promising value of studying changes in DNA methylation and ncRNA expression in various pain conditions is that they can be used as biomarkers of pain state and progression. The ability of ncRNAs to convey themselves to the circulation makes them easily detectable in patients. Therefore, efforts should be taken towards standardising those measurements in order to assure that they are useful tools for diagnosis and prognosis. The efforts should include calibration of circulating levels of ncRNAs in health and disease, the type of samples used for analyses (pre-miRNAs in the nuclei vs mature miRNA elsewhere) and methods of analysis, among others.

## Figures and Tables

**Table 1:** Methylated DNA and ncRNAs involved in nociceptive processing.

Epigenetic Mechanism	Epigenetic Mark/Enzyme	Animal Model	Pain Model	Site	Behaviour Assessed	Targets (Genes or Enzymes)	References
DNA Methylation	(p)MeCP2	Rat	CCI	SC neurons	Thermal, mechanical	-	[17]
			CFA injection in ankle joint		Mechanical	*SGK1*, *FKBP5*, *Sin3a*	[18]
			CFA		Thermal, mechanical	*Zif268*, *Fos*	[19]
			CFA injection in ankle joint, SNI		-	-	[20]
		Mouse	SNI	SC neurons, DRG	Thermal, mechanical	*miR-132*, *CREB*	[21]
	DNMT1	Mouse	SNL	DRG	Thermal, mechanical, cold	*Kcna2*	[22]
	DNMT3a	Rat, mouse	SNL, CCI, CFA	DRG	Thermal, mechanical, cold	*Kcna2*	[23]
		Rat	SNL	SC neurons	Thermal, mechanical	*CSF1*	[24]
		Mouse	Partial SNL	Amygdala	Thermal, mechanical, stress	-	[25]
	MBD1, DNMT3a	Mouse	SNL, CFA	DRG	Thermal, mechanical, cold, capsaicin	*Oprm1*, *Kcna2*	[26]
	DNMT3a2	Mouse	CFA	SC neurons	Thermal, mechanical	*Ptgs2*	[27]
	DNMT3b	Rat	CFA	DRG	-	*CXCR4*, *NFkB*	[28]
					Thermal, mechanical	*miR-29B*	[29]
		Mouse	SNL	SC neurons	Thermal, mechanical	*CXCR3*, *C/EBPα*	[30]
						*GPR151*	[31]
	Wnt3a	Rat	CCI	SC neurons	Thermal, mechanical	*Wnt3a*	[32]
	Tet1	Rat	SNL	SC neurons	Mechanical	*BDNF*	[33]
				DRG	Thermal, mechanical	*Oprm1*, *Kcna2*	[34]
			CFA	SC neurons	Thermal, mechanical	*mGluR5*	[35]
	Tet1, Tet3, 5hmC	Mouse	CFA	SC neurons	Thermal, mechanical	*Stat3*	[36]
	Promoter de-/hyper-methylation	Rat	CFA	DRG	Mechanical	*CBS*, *MBD4*, *Gadd45α*	[37]
			CCI	SC	-	*Wnt3a*	[32]
			SNI	PFC, T cells	Mechanical	*Pak1*, *Pax6*, *Clip3*, *Srp54a*, *Xxpo4* and others	[38]
			SNL	DRG	-	*AFT3*, *A2m*, *NGF*, *SOCS3*, *SOX11*, *STAT3* and others	[39]
		Mouse	CFA	SC neurons	Thermal, mechanical	*miR-219*	[40]
			SNI	PFC,	Mechanical	*Syt2*	[41]
		Mouse and Human	Aging, low back pain	IVD	-	*SPARC*	[42]
	Promoter de-/hyper-methylation	Human	-	Blood tissue	Preoperative and chronic pain	*TRPA1*	[43]
					Crohn’s disease		[44]
					Chronic nociceptive pain vs. chronic neuropathic pain	*RAB10*, *BMP1*, *LRRC59*, *PNPLA6*, *P3H3* and others	[45]
					Chronic lower back pain	*CELSR1*, *KIF11*, *MINK1*, *NAV1* and others	[46]
				Blood tissue and multiple brain regions	Discordant heat pain sensitivity	*TRPA1*, *ST6GALNAC3*, *MICAL2* and others	[47]
lncRNA *	DLEU1	Rat	CCI	SC	Thermal, mechanical	miR-133a-3p/SRPK1	[48]
	DS-lncRNA	Mouse	CCI, SNL	DRG	Thermal, mechanical	-	[49]
	MALAT1	Rat	CCI	SC	Thermal, mechanical	miR-129-5p/HMGB1	[50]
	NEAT1	Rat	SCI	SC	Thermal, mechanical	miR-128-3p/AQP4	[51]
	145 up- and 267 down-regulated lncRNAs	Rat	Paclitaxel-induced peripheral neuropathy	SC	-	Multiple targets	[52]
circRNA *	Circ_0005075	Rat	CCI	SC	Thermal, mechanical	miR-151a-3p/NOTCH2	[53]
	circSMEK1	Rat	CCI	SC microglia	Thermal, mechanical	miR-216a-5p	[54]
	374 different circRNA	Rat	CCI	DRG	-	Dopaminergic synapse, renin secretion, MAPK pathway and neurogenesis	[55]
	circSlc7a11 and 7 others	Rat	Model of bone cancer pain	SC	-	Altered cell proliferation and apoptosis	[56]
	zRANB1	Rat	CCI	SC	Thermal, mechanical	miR-24-3p/LPAR3, Wnt5a/β-catenin pathway	[57]
	134 lncRNA, 12 miRNA and 188 circRNA	Rat	SNI	SC	-	Multiple pathways	[58]
	9 lncRNA, 148 miRNA and 135 circRNA	Mouse	Model of diabetic neuropathic pain	SC	Mechanical	Multiple pathways	[59]
miRNA *	72 different miRNA	Rat	SNI	DRG	Thermal, mechanical	17,316 target genes	[60]
	miR-10a, −29a, −98, −99a, −124a, −134, -183	Rat	CFA injection to masseter muscle	Trigeminal ganglia	-	-	[61]
	miR-103	Rat	SNL	SC neurons	Thermal, mechanical	*Cacna1c*, *Cacna2d1 Cacnb1*	[62]
	miR-103a-3p, -107	Human	Fibromyalgia	White blood cells	Coping strategy	Multiple genetic targets (in silico)	[63]
	miR-107	Rat	CFA	SC	Mechanical	*Slc1a2* (glutamate transporter 1, GL-1)	[64]
	miR-124	Mouse	Model of cancer pain	SC	-	*Synpo*	[65]
	miR-124-1, -33, -380	Mouse	Model of diabetic peripheral neuropathy	DRG neurons	Mechanical	*Kcnab2*, *Serpinb6a*, *Emb*, *Pacsin1* and others.	[66]
	miR-124a	Mouse	Formalin Injection	SC neurons	Formalin	*MeCP2*	[67]
	miR-132-3p, -146b-5p, -155-5p, -384	Human	Trigeminal neuralgia	Serum	-	-	[68]
	miR-134	Rat	CFA	DRG	Thermal, mechanical	*Oprm1*	[69]
	miR-135-5p	Mouse	Model of bone cancer pain	SC astrocytes	Mechanical, spontaneous flinching	*JAK2/STAT3 signalling*	[70]
	miR-137	Rat	CCI	DRG neurons, SC	Thermal, mechanical	*Kcna2* (Kv1.2)	[71]
	miR-140	Rat	CCI	DRG	Thermal, mechanical	*S1PR1*	[72]
	miR-145-5p, -341, -300-5p, -653-5p	Rat	CCI vs. CCI-exercise	DRG	Thermal, mechanical	Multiple targets	[73]
	miR-155, miR-223	Mouse	Facial carrageenan injection	Prefrontal cortex	Mechanical	*C/EBPβ*, *GCSF*	[74]
	miR-183	Mouse	Model of osteoarthritis	DRG	Weight distribution between paws	*TGFα*, *CCL2/CCR2 signal*	[75]
		Human	Patients with osteoarthritis pain	Joint fluid (serum)	-	*-*	
	miR-21	Rat	SNL, CCI	DRG	Thermal, mechanical	*-*	[76]
	miR-214-3p	Rat	SNL	SC neurons	Thermal, mechanical	*CSF1*	[24]
	miR-216a-5p	Rat	CCI	SC	Thermal, mechanical	*IL-6*, *TNF-α*, *IL-1β*, *KDM3A and Wnt/β-catenin pathway*	[77]
	miR-219	Mouse	CFA, formalin injection	SC neurons	Thermal, mechanical, formalin	*CaMKIIγ*	[40]
	miR-221	Rat	Model of diabetic peripheral neuropathy	Serum exosomes from blood samples	Thermal, mechanical	*SOCS3*	[78]
	miR-223	Mouse	CCI	SC	Thermal, mechanical	*NLRP3/IL-1* *β pathway*	[79]
	miR-30a-3p	Rat	CCI	SC	Thermal, mechanical	*EP300*, *bdnf*	[80]
	miR-30b-5p	Rat	Model of osteoarthritis	Cartilage	-	*NF-κB*	[81]
		Human	Patients	Cancerous tissues			
	miR-30c-5p	Mouse, rat	SNI	CSF, DRG, plasma, SC	Mechanical	*TGF-β1*	[82]
		Human	Ischemic neuropathic pain	CSF, plasma	Severity of neuropathic pain	-	
	miR-330	Mouse	Model of pancreatic cancer pain	SC	Mechanical (abdominal)	*Gababr2*	[83]
	miR-34c-5p	Mouse	Model of bone metastatic pain	DRG	-	*Cav2.3*, *P2rx6*, *Oprd1*, *Oprm1*	[84]
	miR-375	Mouse	Morphine analgesic tolerance	DRG	Thermal	*Janus kinase 2 (JAK2)*	[85]
	miR-485-5p	Rat	CFA	DRG	Mechanical	*Asic1*	[86]
	miR-590-3p	Mouse	Model of diabetic peripheral neuropathy	DRG	Thermal	*RAP1A*	[87]
	miR-7a	Rat	SNL, CCI, CFA	DRG	Thermal, mechanical	*Scn2b*	[88]
	miR-96	Rat	CCI	DRG	Thermal, mechanical	*Nav1.3*	[89]
piRNA	piRNA-DQ541777	Mouse	CCI, CFA	SC neurons	Thermal, mechanical	*Cdk5rap1*	[90]

* Note there are other ncRNA involved in nociceptive processing, which are not included in this table as have been covered in recent reviews, see [91,92,93,94,95].

**Table 2:** Techniques used to study DNA methylation.

Techniques	Mechanism of Action	Strength	Weakness	Resolution	Cost	References
**Sodium bisulphite sequencing:** Conversion of unmethylated cytosine residues to uracil then to thymine. Gold standard technique.						
RRBS	Use of restrictions enzymes to enrich CpG sites, stabilising methylated sites.	Genome wide coverage. High sensitivity. More cost effective than WGBS.	Cannot identify between 5mC and 5hmC. Significant degradation and fragmentation of DNA segments.	Single base	Moderate (~£200–£500)	[45,123]
T-WGBS	Gold standard assaying technique. Genome wide analysis of methylated sites.	Genome wide coverage of majority of CpG sites to evaluate methylation.	Cannot identify between 5mC and 5hmC. Significant degradation and fragmentation of DNA segments.	Single base	High (~£700–£3000)	[124]
TAB-seq	Oxidation of TET proteins combined with 5mC to localise 5hmC.	Protein able to differentiate between 5mC and 5hmC.	Significant degradation and fragmentation of DNA segments. Conversion using TET enzymes disrupts sequence alignment, thus unmethylated residues may remain. High depth sequencing required to detect low abundance 5hmC.	Single base	High (~£1000)	[125,126]
**Differential enzymatic cleavage of DNA:** Enzymatic restriction of DNA resulting in methylated CpG fragments.						
DREAM	Enzymatic digestion of DNA through utilisation of restriction endonucleases.	More cost-effective compared to bisulphite conversion. Ability to detect methylated CpG sites at low density levels.	High sensitivity. Cost-effective compared to sodium bisulphite sequencing techniques.	High	Moderate	[126,127]
**Affinity capture of methylated DNA:** Use of methyl- CpG-binding domain (MBD) proteins or MeDIP to bind to methylated DNA.						
MBDCap-Seq	Methy-CpG binding domain based (MBD) protein captures DNA methylation, identifying highly differentiated regions.	Greater sensitivity than MeDIP in high density CpG sites. Protein able to differentiate between 5mC and 5hmC. Higher sensitivity than MeDIP in higher density CpG regions.	Resolution lower than other techniques. Sensitive to hypermethylated regions.	150 bp	Moderate (~£100)	[123]
MeDIP	Methylated DNA immunoprecipitation uses antibodies specific to 5mC to precipitate methylated DNA.	More sensitivity than MBDCap-Seq in low density regions.	Sensitive to hypermethylated regions. Cannot predict absolute methylation.	100 bp	Moderate (~£100)	[128]

Many of these techniques, such as T-WGBS, require multiple samples to be analysed for sequencing; therefore, while cost effectiveness is approximately quantified, this is an estimation and will therefore vary on the size and type of genomic material. DMR: differentially methylated region. DREAM: digital restriction enzyme analysis of methylation. DRG: dorsal root ganglion. MBDCap-Seq: methyl-CpG binding domain-based capture and sequencing. MeDIP: methylated DNA immunoprecipitation sequencing. RBBS: reduced representation bisulphite sequencing. TAB-seq: TET-assisted bisulphite sequencing. TET: ten-eleven translocation. T-WGBS: tagmentation-based whole-genome bisulphite sequencing.

## Data Availability

Not applicable.

## Abbreviations

8-oxoG: 8-oxoguanine; A2bp1: ataxin 2 binding protein 1; ASIC: acid-sensing ion channel; Bdnf: brain-derived neurotrophic factor; CaMKIIγ: calcium/calmodulin-dependent protein kinase II γ; Cav1.2-LTC: Cav1.2-comprising L-type calcium channel; Cav2.3: calcium voltage-gated channel subunit alpha1 E, or CACNA1E; CBS: cystathionine-β-synthase; CCI: chronic constriction injury of the sciatic nerve; Cdk5rap1: CDK5 regulatory subunit-associated protein 1; CEBP: CCAAT enhancer binding protein; CFA: complete Freund’s adjuvant; Ch/GCE: Choline monolayer-modified glassy carbon electrode; circRNA: circular RNA; circSMEK1: circular RNA SMEK1; Clcn3: chloride voltage-gated channel 3; CNS: central nervous system; COMT: catechol o-methyltransferase; Cox-2: cyclooxygenase 2; CSF: cerebrospinal fluid; CSF1: colony-stimulating factor-1; CXCL10: c-x-c motif chemokine ligand 10; CXCR3: c-x-c chemokine receptor type 3; CXCR4: c-x-c chemokine receptor type 4; DLEU1: deleted in lymphocytic leukemia 1; DNMT: de novo DNA methyltransferases; DREAM: digital restriction enzyme analysis of methylation; DRG: dorsal root ganglia; DS-lncRNA: DRG-specifically enriched lncRNA; EHMT2/G9a: Euchromatic histone-lysine N-methyltransferase 2; Fkbp5: FKBP prolyl isomerase 5; GABA: gamma aminobutyric acid; GABABR2: GABA beta receptor 2; Gadd45: growth arrest and DNA damage inducible protein Gadd45; GCSF: granulocyte colony-stimulating factor; GLT-1: glutamate transporter 1; gp120: glycoprotein 120; GPCR: G-protein-coupled receptor; GPR151: G-protein-coupled receptor 151; HMGB1: High mobility group box 1; HPLC: High-Performance Liquid Chromatography; IKBKB: inhibitor of NF-κB kinase subunit beta; JAKs/Stat3: Janus Kinases/Signal transducer and activator of transcription protein 3; Kcna2: potassium voltage gated channel subfamily A member 2; Kv1.2: potassium voltage gated channel subfamily A member 2, encoded by Kcna2 gene; lncRNA: long ncRNA; LUMA: Luminometric Methylation Assay; MALATI: metastasis associated lung adenocarcinoma transcript 1; MBD: methyl-CpG-binding domain; MBD1: methyl CpG binding domain protein 1; MBD4: methyl CpG binding domain protein 4; MeCP2: methyl CpG binding protein 2; MeDIP-Seq: Methylated DNA immunoprecipitation followed by sequencing; Mef2c: myocyte enhancer factor 2C; mGluR5: metabotropic glutamate receptor subtype 5; miRNA: micro-RNA; MREs: methylation-sensitive restriction enzymes; mRNA: messenger RNA; mtDNA: mitochondrial DNA; MWCNTs: multi-walled carbon nanotubes; Nav1.3: voltage-gated sodium channel type III alpha subunit; ncRNA: non-coding RNA; NEAT1: nuclear paraspeckle assembly transcript 1; NGF: nerve growth factor; notch2: notch receptor 2; OGG1: enzyme 8-oxoG glycosylase-1; Oprd1: opioid receptor delta 1; Oprk1: opioid receptor kappa 1; Oprm1: opioid receptor mu 1; P2rx6: purinergic receptor P2X6; PCR: polymerase chain reaction; piRNA: piwi-interacting RNA; PLGA: poly lactic-co-glycolic acid; Pou4f3: transcriptional activator POU domain, class 4, transcription factor 3; PPyox: polypyrrole; Ptgs2: prostaglandin endoperoxide synthase 2; PTM: post translational modifications; RALY: RALY Heterogeneous Nuclear Ribonucleoprotein; RNA-Seq: high throughput RNA sequencing; S1PR1: sphingosine-1-phosphate receptor 1; SAM: S-adenosyl methionine; Sgk1: serum/glucocorticoid regulated kinase 1; Sin3a: SIN3 transcription regulator family member A; siRNA: short interfering RNA; SNI: spared nerve injury; SNL: spinal nerve ligation; SPARC: secreted protein, acidic rich in cysteine; SRPK1: SRSF Protein Kinase 1; Sst: somatostatin; STAT3: signal transducer and activator of transcription 3; Synpo: synaptopodin; syt2: synaptotagmin 2; TDG: thymine DNA glycosylase; TET: ten-eleven translocation family; Tet1: Tet methylcytosine dioxygenase 1; TGF-β: transforming growth factor-beta; TRP: transient receptor potential channels; TRPA1: TRP ankyrin 1; Wnt3a: wingless related integration site 3a.
